# Safety and Danger Considerations of Novel Treatments for Atopic Dermatitis in Context of Primary Cutaneous Lymphomas

**DOI:** 10.3390/ijms222413388

**Published:** 2021-12-13

**Authors:** Karol Kołkowski, Magdalena Trzeciak, Małgorzata Sokołowska-Wojdyło

**Affiliations:** 1 Dermatological Students Scientific Association, Department of Dermatology, Venereology and Allergology, Faculty of Medicine, Medical University of Gdansk, 80-214 Gdansk, Poland; 2 Department of Dermatology, Venereology and Allergology, Faculty of Medicine, Medical University of Gdansk, 80-214 Gdansk, Poland; mtrzeciak@gumed.edu.pl (M.T.); malgorzata.sokolowska-wojdylo@gumed.edu.pl (M.S.-W.)

**Keywords:** cutaneous lymphoma, mycosis fungoides, Sézary syndrome, cytokine, atopic dermatitis, tumor microenvironment, biologic treatment, small molecule inhibitors, JAK-STAT pathway, interleukins

## Abstract

The impact of new and emerging therapies on the microenvironment of primary cutaneous lymphomas (PCLs) has been recently raised in the literature. Concomitantly, novel treatments are already used or registered (dupilumab, upadacitinib) and others seem to be added to the armamentarium against atopic dermatitis. Our aim was to review the literature on interleukins 4, 13, 22, and 31, and JAK/STAT pathways in PCLs to elucidate the safety of using biologics (dupilumab, tralokinumab, fezakinumab, nemolizumab) and small molecule inhibitors (upadacitinib, baricitinib, abrocitinib, ruxolitinib, tofacitinib) in the treatment of atopic dermatitis. We summarized the current state of knowledge on this topic based on the search of the PubMed database and related references published before 21 October 2021. Our analysis suggests that some of the mentioned agents (dupilumab, ruxolitinib) and others may have a direct impact on the progression of cutaneous lymphomas. This issue requires further study and meticulous monitoring of patients receiving these drugs to ensure their safety, especially in light of the FDA warning on tofacitinib. In conclusion, in the case of the rapid progression of atopic dermatitis/eczema, especially in patients older than 40 years old, there is a necessity to perform a biopsy followed by a very careful pathological examination.

## 1. Introduction

Primary cutaneous lymphomas (PCLs) are a rare entity of lymphoproliferative disorders that have no evidence of extracutaneous involvement at the time of diagnosis [1]. An important impact of the tumor microenvironment on the progression of the disease has been raised in literature [2]. Currently, a variety of drugs affecting the cytokines and pathways are essential in the pathogenesis of atopic dermatitis (AD) and are in the clinical trials phase, whereas dupilumab targeting interleukin-4 (IL-4) and interleukin-13 (IL-13), tralokinumab targeting IL-13 and two Janus kinase inhibitors (JAKi): upadacitinib (JAK1 inhibitor) and baricitinib (JAK1/JAK2 inhibitor), are already registered in the EU [3,4]. Agents blocking interleukin-22 (IL-22) and interleukin-31 (IL-31), fezakinumab, and nemolizumab, as well as lebrikizumab will be available for patients soon [3]. There is a controversy regarding a potential of increased risk of lymphoma in patients with atopic dermatitis (AD). Our aim is to elucidate the role of IL-4, IL-13, IL-22, IL-31, and the JAK/STAT pathway in PCLs in the context of novel treatment of AD.

## 2. Discussion

AD is a chronic, inflammatory skin disease characterized by strong pruritus that less commonly affects adults [5]. This condition is associated with a poorer quality of life in comparison with the general population and causes sleep disturbances and coexisting comorbidities [6]. As reported by the epidemiological studies, the prevalence of the childhood AD is between 12% and 20% in the United States, Europe, and Eastern Asia, whereas in the elderly population it ranges from 2% to 5% [7,8,9,10,11,12]. Moreover, the secular trends tend to show an increase in the number of AD patients in both children and adults [9,10]. Unfortunately, a significant number of these patients present moderate to severe AD. Despite the scale of the problem, the arsenal of drugs with a safe profile of action, characterized by a low risk of serious side effects, and appropriate for long-term use is scarce [13]. Therefore, doctors and patients hope for the end of “the draught”, which may happen thanks to biologic drugs, e.g., monoclonal antibodies (mAb) like dupilumab/tralokinumab or small molecule inhibitors, e.g., upadacitinib/baricitinib, which are proven to be effective and are registered in the EU [13]. In fact, a few of these medications are already approved for topical and systemic treatment of AD. However, despite the unquestionable potential these drugs hold for AD patients in relieving their burden, we believe that some important issues must be raised.

Among PCLs, heterogenous groups of B-, T- and NK-Cell lymphomas have been differentiated [1]. Mycosis fungoides (MF) belongs to cutaneous T-cell lymphoma (CTCL) and its classical variant is the most common PCL [1]. Our review focuses on the CTCLs; however, when PCLs are mentioned, we refer to the entire spectrum of primary cutaneous lymphomas. Major meta-analysis has shown a relative risk ratio (RR) of developing a lymphoma of 1.43 (95% CI, 1.12–1.81) in patients with AD [14]. The risk of lymphoma is higher in cases where highly potent TCSs are used and in a severe course of the disease [14]. In a recent study, the hazard ratios of developing Non-Hodgkin’s lymphoma (NHL) increase with the severity of the eczema [15]. This was the only epidemiological study in which we could find any biologic drug taken into consideration. Dupilumab has been analyzed in the Danish cohort together with the influence of other immunosuppressive drugs, including cyclosporine, azathioprine, mycophenolate and methotrexate [15]. According to some studies, the risk of developing NHL with cutaneous manifestation is especially high, but we have to bear in mind the possible misdiagnosis bias [14,15,16]. We were not able to find any other studies that describe the incidence of lymphomas in patients treated with biologics or small molecule inhibitors referring to AD except clinical trials and case reports. Incidence of lymphomas in the mentioned studies will now become a baseline for the further analysis of the effects of new immunosuppressives brought to the market.

It may be difficult to clinically differentiate AD and PCL, especially in the case of erythroderma. If a patient develops adult-onset AD, erythrodermic CTCL, and Sézary syndrome (SS), they should always be excluded, as these diseases require distinct treatment and have drastically varying prognoses [17]. Similarities concerning both diseases, which are crucial in their pathogenesis, are illustrated in the Table 1 [17,18,19,20,21,22,23,24,25,26,27,28].

Some authors indicate that, due to the significant quantity of similarities, both diseases may require the same treatment at certain stages [29]. However, in our opinion, the safety of emerging drugs used in AD treatment, in the context of a PCL coexistence/induction risk, should be raised. We decided to analyze theoretical and clinical data regarding interleukins and JAK-STAT pathways, which recently have been proven to be attractive targets in the treatment of AD. On that basis, we excluded IL-5, IL-17, and IL-33 from the analysis, as the trials of drugs targeting them are either terminated, are of unknown status, or they did not meet the primary endpoints [30,31,32,33,34,35]. In this review, we also omit the IL-12/IL-23 axis affected by ustekinumab for two reasons. Firstly, a recent review on the effectiveness of this agent concluded that the IL-12/IL-23 pathway is not an attractive target for the treatment of AD [36]. Furthermore, the largest cohort of patients receiving ustekinumab has shown that more novel and effective treatments are available for the therapy of this atopic disease [36]. Second, IL-12 has been shown to be one of the possible treatments, despite the fact that it is not currently clinically developed [37]. Therefore, blocking it should be a factor facilitating the lymphoma progression by down-regulating the Th-1 cytotoxicity against malignant clones.

### 2.1. New Medications in AD

AD is thought to be the hallmark of Th-2 microenvironment diseases. Th-2 profile cytokines, such as IL-4, IL-5 and IL-13, play a significant role in the pathogenesis of the disease by switching the immunoglobulin class to IgE and stimulating afferent neurons via IL-4Rα, thereby promoting pruritus [38]. Therefore, drugs blocking these pathways should be clinically effective in reducing the symptoms of this eczematous disease, as they act against the inflammation [39].

One of them is dupilumab—a fully human monoclonal antibody that blocks IL-4Rα, a shared receptor unit for IL-4 and IL-13, actively participating in the decrease of Th-2 mediated immunological response [3]. It is already used in America, Europe, and in several other countries on children, adolescents, and adults. The analysis of four phase-three trials has revealed that patients treated with this monoclonal antibody achieve a significantly higher percentage reduction from the baseline in the most important AD management scales—Eczema Area and Severity Index (EASI), SCORing Atopic Dermatitis (SCORAD), Dermatology Life Quality Index (DLQI), and Patient-Oriented Eczema Measure (POEM) versus control [40]. Notably, these superior effects have been achieved in monotherapy without topical corticosteroids, regardless of previous use of systemic non-steroidal immunosuppressants, e.g., methotrexate or cyclosporine [40].

Other drugs targeting the IL-13 are lebrikizumab and tralokinumab. IL-13 binds and neutralizes the activity of the mentioned cytokine with high affinity [41]. In phase IIb of several randomized clinical trials, it showed promising results [42,43]. Even though adverse effects of this drug were reported in the significant group of patients, they were mostly mild to moderate [42,43]. Phase III clinical trials on patients who suffer from moderate to severe AD are currently ongoing [44,45,46,47,48,49,50]. Another promising emerging drug is tralokinumab—a fully human, monoclonal anti-IL-13 IgG4 antibody that binds to two subunits of IL-13R (IL-13Rα1 and IL-13Rα2), thus neutralizing the cytokine from the interaction [3,51]. Recently, three phase III clinical trials (ECZTRA1, ECZTRA2, and ECZTRA3) were completed for this drug [52,53]. Tralokinumab, in combination with topical corticosteroids, is not only effective in reducing the pruritus and improving sleep quality, but it is also well tolerated for up to 52 weeks of treatment, which brings a promising perspective we mentioned earlier [52]. Moreover, this medicament is safe and well tolerated in combination with topical corticosteroids [53]. Interestingly, a long-term extension trial for patients who were participants in the previous studies is currently ongoing and the estimated completion date is in 2024 [54].

IL-22 and IL-31 are also the targets of new drugs, which have been or currently still are under investigation in phases IIa and III of clinical trials [55,56,57]. Fezakinumab, an anti-IL-22 antibody, has been shown in the IIa randomized, double-blind clinical trial on adults with moderate to severe AD to be well tolerated and to have sustainable improvements after the last dose [55]. Despite the small sample size and common adverse effects, which were upper respiratory tract infections, improvements in SCOring AD (SCORAD) were significant in patients with severe disease [3,55]. Thus, this drug is thought to be suitable for patients with severe AD, but no further clinical trials are currently ongoing [3]. Another interesting medication, especially for managing the pruritus in patients with AD is nemolizumab, a human monoclonal IL-31 receptor α (IL-31Rα) antagonist [3,57,58]. This drug targets small-diameter neurons and it is thought that the relieving effect of nemolizumab is due to action on cutaneous sensory neurons [3,58]. In the phase III trial, the patients who could not achieve proper control of pruritus by solely using topical treatment were recruited and enrolled [56]. Not only were the primary end points of the study achieved with a significant decrease in pruritus measured in the VAS scale, but also a series of secondary endpoints including EASI, DLQI or Insomnia Severity Index were met [56]. Other phase III trials are currently ongoing [59].

Various systemic and topical JAK inhibitors are about to be widely used in the treatment of AD [4]. The data on the double blind control trials evaluating the efficacy of these drugs in the treatment of AD are promising [60,61]. Baricitinib, abrocitinib, and upadacitinib belong to the group of oral drugs, while ruxolitinib is known as a topical agent considered in the therapy of AD [60].

Baricitinib is known as the first-generation JAK1/2 selective inhibitor [60,62,63]. The efficacy of the drug in monotherapy and combined with topical corticosteroids has been evaluated and the dose of 4 mg appears to significantly improve symptoms [64,65,66]. In the pooled safety analysis of baricitinib in adults, which contained previously mentioned studies, there were four major cardiovascular-adverse events and one death, however, no malignancies were reported [67].

Abrocitinib is an oral selective JAK1 inhibitor that achieved satisfying results in the phase III trial, proving that it is effective and well tolerated in monotherapy [64,68]. Patients from these studies have been enrolled in the extended trial (NCT03422822) and in the 48th week of this trial, it has been shown, that between 24 and 36 weeks, the proportion of patients meeting primary endpoints increased and was stable thereafter [60]. Comparing abrocitinib, dupilumab, and the placebo in clinical trial, both drugs significantly more reduced AD symptoms; however, a 200 mg dose of abrocitinib was superior to dupilumab in limiting itchiness [69].

The next oral selective JAK1 inhibitor is upadacitinib [60]. It safe and efficient in the monotherapy of moderate to severe AD in three phase III trials [70,71]. Moreover, in comparison with dupilumab it was superior, showing significantly higher proportion of patients who achieved the primary and secondary endpoints of the study [72]. Extension of the mentioned trials and also new ones with pediatric patients are ongoing [73,74].

Ruxolitinib (JAK1/2 inhibitor) and delgocitinib (pan-JAK inhibitor) have proved to be effective topical drugs in AD [60,61]. In the two phase III trials, ruxolitinib has shown anti-inflammatory and anti-pruritic effects superior to the vehicle cream [75]. Adverse effects were infrequent and clinically insignificant [75]. Clinical trials with atopic children are underway [76]. Delgocitinib also seems to be satisfactory, since in the phase III trial it was effective and well tolerated in Japanese patients for up to 28 weeks [77]. Currently, two phase III trials on moderate to severe chronic hand eczema are ongoing [78,79].

### 2.2. Role of Interleukin-4 and Interleukin-13 in PCL

Interleukin-4 (IL-4) and interleukin-13 (IL-13) are the characteristic molecules that induce, drive, and prolong the Th-2 answer both in AD and in advanced stages of CTCL [21,22,23,38]. Along with the progression of CTCL, Th-2 cytokines are most commonly overproduced, skewing the Th1/Th2 axis towards the latter side, a well-known phenomenon [80,81,82]. Certain genetic markers are associated with the predisposition to develop the progressive MF. One of them has an increased expression of IL-4 relative to CD3 expression levels, which are significantly associated with lymphoma progression [83]. Each of the discussed cytokines are elevated in the biopsies of MF and SS lesions [84,85,86,87]. IL-4 levels are raised in sera of patients; however, it does not always concern the cases of low-grade lymphoma [18,21,81,82,84,88]. The elevated concentrations of these cytokines are related to the ability of neoplastic cells to secrete IL-4 and IL-13 as well as in the skin and in the blood in vivo [81,82,85].

Also, IL-4 is a potent factor that polarizes tumor-associated macrophages into type 2 cells (M2 Macrophages) [89]. The ability to produce several Th-2 cytokines is characteristic for these phagocytes, thereby affecting the formation of CTCL by stromal factors [86,89]. Furthermore, IL-4 and IL-13 are important growth factors for PCLs and IL-13 acts in an autocrine manner on the neoplastic lymphocytes [81,87]. IL-4 and IL-33 are also found to induce the secretion of IL-31, which is one of the elements alongside with the discussed interleukins, causing pruritus in AD and in CTCL—but the data are ambivalent concerning lymphomas [3,90]. These properties contribute to driving the Th-2 type inflammatory answer propelled in a vicious circle, leading to the depletion of the Th-1 microenvironment. The arrest of the IL-4/IL-13 pathway by neutralizing IL-4 and IL-13 cytokines leads to inhibition of tumor-cell proliferation [81]. Interestingly, blocking certain types of IL-13 receptors (IL-13Rα2) revealed an even stronger inhibition effect. However, this receptor binds with IL-13 stronger than the first IL-13 receptor (IL-13Rα1) and is thought to be a decoy in the normal tissues [81,91]. Recent studies have shown that the tumoral microenvironment created by the malignant lymphocytes in leukemic CTCL is a global bias, which refers also to benign T cells [82]. Thus, in comparison to the normal lymphocytes in healthy individuals, non-tumorous cells are strongly Th-2 biased [82]. Such drastic reduction of the cytotoxic environment is thought to be one of major factors leading to infections which is the most common reason of death in this group of patients [1,92,93].

It is also proven that cytoplasmic IL-4 concentration is the predictor of the advanced stage of MF and SS [94]. Moreover, increased IL-4 concentration is observed frequently in advanced stages of CTCL; it correlates with T-cell immunophenotype differences found in advanced lymphoma stages and is associated with clonality of MF and SS cells [94]. Some available methods of treatment used in clinical practice can reduce the Th-2 polarization in advanced stages of CTCLs. One study revealed that extracorporeal photopheresis (ECP) effectively restores the imbalance in Th1/Th2 microenvironments of peripheral blood mononuclear cells (PBMC) [95]. After one year of such therapy, the concentrations of IL-4, interferon gamma (IFNγ), and IL-12 did not differ from the healthy controls [95]. Also, after administration of the T-cell depleting antibody directed against CD52, alemtuzumab (Campath), which is used to treat refractory leukemic CTCL (L-CTCL), skin T cells have been shown to secrete less IL-4 and more interferon gamma (IFNγ) than before the treatment [96]. Finally, Guenova et al. suggested that inhibiting the Th-2 microenvironment and restoration of Th-1 cytotoxicity should enhance both anti-tumorous and antibacterial responses [82].

Concluding this section, the listed Th-2 cytokines play an essential role in the pathogenesis of PCLs. It is especially prominent in advanced stages of the disease. Therefore, blocking these pathways may be beneficial and may result at least in the stabilization of the lymphoma.

### 2.3. Role of Interleukin-22 in PCL

Interleukin-22 (IL-22) is secreted mainly by the subpopulation of Th22 lymphocytes, but also by other immune cell subsets and is involved in the modification of tissue responses at the inflammation [97]. In two studies and one case report, levels of this cytokine were significantly elevated in both skin lesions and sera of CTCL patients [18,98,99]. Other researchers found that cultured CTCL cells overexpress IL-22 receptor subunit alpha1 (IL22Rα1) and overproduce IL-22 as well as chemokine ligand 20 (CCL20) [100]. Cytokine induces the expression of CCL20 and signal transducer and activator of transcription 3 (STAT3), the latter of plays a role in the pathogenesis of CTCL [98,101]. Moreover, the Sézary cells of one patient who developed sepsis stained positive for CD8 and produced IL-22 [98]. Genetic analysis of SS patients and SS cell lines (SaEx) showed the disruption in the IL-22 receptor subunit alpha2 (IL22Rα2) gene twice [102]. Subsequently, a fusion of several genes occurred and one of them was the CCDC28A-IL22Rα2, which was transcribed on the messenger RNA level [102]. CCL20 and IL-22 serum levels correlate with the LDH and sIL-2R and thus they correlate with CTCL severity [18]. Their activity seems significant in the pathogenesis of the disease.

Enhanced CCL20 activity may induce epidermal hyperplasia as well as the migration of chemokine receptor 6 (CCR6) positive Langerhans/Dendritic cells to the skin, which are crucial in the evolution of lymphoma cells as the activation of T-cell receptors is crucial for the malignant transformation of MF [18,103]. CCL20 is a ligand of CCR6 [100]. Activation of IL-22 has also been shown to lead to chronic CCR6-CCL20 interaction with CTCL cells [100]. Furthermore, the continuous upregulation of CCR6 was discovered, which results in the continuous activation of CCR6-CCL20, leading the lymphoma cells to metastasize to internal organs [100]. These important findings are supported by recent studies, showing that triggering a STAT3/CCL20/CCR6 cascade that blocks CCR6-CCL20 interaction, may be crucial in stopping lymphomagenesis [104]. Today, it is considered one of the promising strategies in the treatment of advanced CTCL [104].

### 2.4. Role of Interleukin-31 in PCL

The role of Interleukin-31 (IL-31) in PCL is difficult to establish despite numerous studies covering this topic. Importantly in other lymphomas, such as follicular lymphoma, IL-31 promotes the growth of tumors in an autocrine and paracrine manner [90]. Certainly, this cytokine seems to be involved in the pathogenesis of PCLs [105]. It is typically secreted by Th-2 cells [106]. Signal transducers and activators of transcription 6 (STAT6) and NF-κB induced by IL-4 are main players in mediating the production of IL-31 [106]. Both STAT6 and NF-κB play some role in the pathogenesis of CTCL [101,107]. IL-31 is elevated in both lesions and sera of patients in the majority of studies (five); however, one study found no differences in comparison to control groups [54,105,108,109,110,111]. Malignant T-cells may secrete IL-31 [109]. Researchers did not establish with certainty whether IL-31 concentration is correlated with CTCL progression or/and pruritus [54,105,108,109,110,111]. Despite the proven central role of this cytokine in mediating pruritus in AD patients and the assumptions to have the same role in CTCL, it is rather not the case here based on our results [58,105,110]. The postulated patomechanism seems to be specific to AD [110].

IL-31, a chemokine ligand expressed by monocytes and dendritic cells, is also correlated with CCL18 and may be associated with the development of CTCL [84]. Both of these cells are important in the pathogenesis of PCL [89,103]. Moreover, exposition of Staphylococcal enterotoxin B, a potent superantigen, to patients with AD rapidly elevates the IL-31 levels secreted by T cells [112]. In cultured patients’ tumor cell samples, IL-2 acted as the previously mentioned superantigen, resulting in the expression of IL-31 in 9 of 11 cases [113]. Both illustrated mechanisms could give a reasonable explanation to the observed elevation of IL-31 levels in patients with PCL. Moreover, it may explain why, in some studies, the concentrations were higher in advanced stages of the disease.

### 2.5. Role of JAK-STAT Pathways in PCL

As of today, four types of Janus kinases (JAKs) (JAK1, JAK2, JAK3 and TYK2) and seven different signal transducers and activators (STATs) (STAT1, STAT2, STAT3, STAT4, STAT5a and STAT5b, STAT6) have been identified [114,115]. These molecules have important roles in the transmission of the cytokine signal in various human cells in vivo [114]. They are abundantly expressed in the healthy human epidermis [115]. Numerous studies have shown the impact of different JAK/STAT pathways on the pathogenesis of PCLs [90,101,103,116,117,118,119,120,121,122,123,124,125,126,127,128,129,130,131,132,133,134,135,136,137,138,139,140,141,142,143,144,145,146,147,148,149,150,151,152,153,154,155,156,157,158,159,160,161,162,163,164,165,166,167,168,169,170,171,172,173,174,175,176,177,178,179,180,181].

Strong evidence has been collected on genetic abnormalities of JAKs and STATs in cutaneous lymphoma cells, both on the cell lines and PBMCs from patients [103,125,129,137,139,143,144,149,153,157,162,170]. Studies highlight the activating mutations of JAK3, which occurred in 3.3–10.8% of patients with PCL according to different study groups [125,137,139]. Suppressors of cytokine signaling 1 (SOCS-1), a potent CTCL suppressor, regulates the JAK3/STAT5 signaling [119,143,153]. Moreover, mutations of SOCS-1 that abolished its binding to JAK3 reinforced the aggressive course of the lymphoma [143]. Other researchers found that SOCS-1 deletion was one of the most common events in the group they studied and happened especially in the early stages of MF [153]. Interestingly, JAK3 is activated by the IL-2 and is in its pathway, which may be clinically relevant especially in more aggressive types of the disease [139]. JAK3 is expressed in the nuclei of CTCL cells, both from cell lines and PBMCs, and may also play a novel role in malignant clones [167]. Interestingly, tofacitinib (JAK1 and JAK3 selective inhibitor) could not block the kinase activity inside the nucleus, in contrast to the normal blockage of the IL-2/JAK3 pathway [167,177].

Other genetic studies regarding PCL found the pathological variants in JAK1 and JAK2 genes only in individual cases, and concerning common JAK2 alterations detected in other lymphoid malignancies are worth noticing [129,137,162,170,176]. Interestingly, JAK2 may play a role in keeping the Th-1 cytotoxic answer against the tumor present by mediating the IL-12 signaling and thereby phosphorylating STAT4 [135]. STAT4 and STAT6 genes are inversely regulated in CTCL and the loss of expression of the former may play a role in switching to Th-2 answer in the advanced lymphoma stages [131]. In contrast, JAK2 inhibition resulted in a decreased viability of SaEx, suggesting that it may be important for tumorous survival [164]. Concomitantly, with these results acquired from cell lines, the PBMC isolates showed that JAK inhibition potentiates the cytotoxicity of other agents (e.g., histone deacetylase inhibitors (HDACi)) and allows us to achieve a more generalized lethal effect against the malignant clones [165]. Other studies seem to be consistent with these results by showing the results of combinative cytotoxic effect of ruxolitinib (selective JAK1 and JAK2 inhibitor) and reminostat (HDACi) on the CTCL cell lines [168]. Moreover, another study showed the synergistic role of JAK/STAT inhibition on the in vivo SS model, in which romidepsin (HDACi) and mechloretamine were successfully used in the treatment [173]. A blockage of the malignant cell growth mechanisms in CTCL after the administration of increasing doses of ruxolitinib has also been suggested [137]. Further use of JAK inhibitors in the treatment of SS may be due to their ability to stop the constitutive activation of the activated kinases [176]. In advanced stages of a lymphoma, STAT3 and STAT5 are completely dependent on the constitutively activated JAK1 and JAK3 [135]. However, one study showed that apoptosis in CTCL lines may be augmented via the JAK1 pathway and this activity was blocked by ruxolitinib [145].

The further parts of the JAK/STAT pathway, especially STAT3, STAT5, and STAT6 also play the established role of mediators in the PCLs oncogenesis [101,157,173,175]. These proteins act upon the regulation of several gene transcriptions after being phosphorylated by JAKs. One study demonstrated the constitutive activation of STAT5 in CTCL; however, in most cases, after blocking the specific Janus kinase, these molecules should not be able to maintain their function in regulating different gene transcriptions [134,135]. Currently, there are no ongoing and upcoming trials concerning AD and STATs inhibition. Therefore, the STATs role in PCLs is beyond the scope of this review.

### 2.6. Safety and Danger Concerns of Administering the New Drugs in the Context of PCLs

AD and psoriasis are distinct medical conditions. However, we have to think about the possible interactions between drugs modifying immune responses in AD and pathogenesis of PCL (followed by Dequidt et al.), especially because of the higher risk of lymphoma in severe cases of AD as well as the problem of differentiation between AD and PCL in some cases [182]. Also, in our opinion, it is crucial to first deduce whether the biologic and small molecule treatments in AD may induce lymphoma and second, in case of an overlap or misdiagnosis between those two diseases, a PCL may progress after administration of these agents.

**Figure 1:**
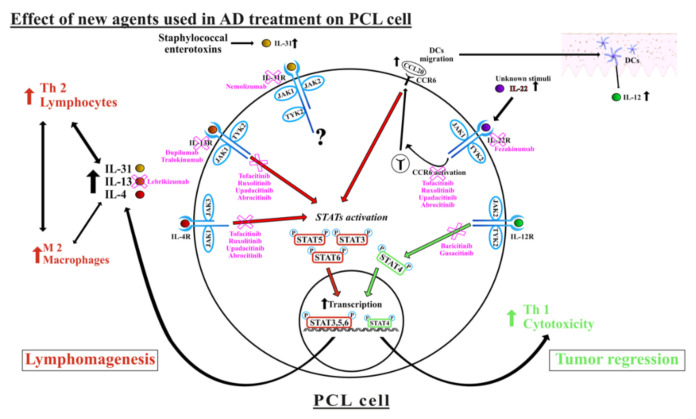
The influence of agents targeting interleukins (IL) 4, 13, 22, and 31 and JAK/STAT pathways on the primary cutaneous lymphomas (PCLs) cells and tumorous microenvironment. The up and down arrows stand for increase/decrease of the interleukins concentration, cell count or receptor’s upregulation. IL-12 promotes phosphorylation of STAT4, thereby stimulating the cytotoxic mediated CD8(+) answer. Concomitantly, IL-4, IL-13, and IL-31 contribute to forming the Th-2 cytokine profile, which results in decreased cytotoxic immunosurveillance and lymphomagenesis. IL-4, IL-13, and IL-22 activate different Janus kinases, which promote the STAT3, STAT5, and STAT6 activation contributing to the transcription of pro-tumorous factors. In the advanced stages of the disease, this phenomenon may be seen more prominently. By blocking several pathways or cytokines, biologic drugs and small molecule inhibitors may affect both the malignant microenvironment and pathways in the PCLs cells.

We would like to shortly summarize the above theoretical assumptions. Decreasing the concentration and/or stopping the secretion of IL-4 and IL-13 could lead to the restoration of the Th-1 microenvironment, which may enhance tumorous toxicity. The reduction in the levels of these interleukins after receiving certain treatments discussed earlier is one of the supporting facts for this theory. Therefore, dupilumab, lebrikizumab, and tralokinumab may appear to be clinically efficient in the treatment of the PCL. Agents blocking IL-22, i.e., fezakinumab, could also stop the lymphomagenesis and additionally reduce the ability of the tumorous cells to metastasize in the advanced stages of the lymphoma. We also show the possible involvement of IL-31 in the pathogenesis of PCLs, which is still elusive. Theoretically, blocking the role in the establishment of the Th-2 microenvironment and in the growth of the tumor might be beneficial for the lymphoma patients after administration of nemolizumab, similar to other lymphomas. Lastly, we described the current state of knowledge on the influence of JAKs on PCLs. JAK1 and JAK3 seem to have the pathogenic role by activating the STAT3, STAT5, and STAT6, which contribute significantly to lymphomagenesis. Therefore, blocking them may reduce tumor development. In contrast, JAK2 may also play some role in preventing the growth of lymphomas. Despite the mentioned effects of ruxolitinib on the CTCL cell lines, obstructing this pathway may appear to be harmful for the patients by reducing the Th-1 cytotoxicity directed to the clones. Figure 1 summarizes the most important aspects of the above assumptions.

However, despite the theoretical expectations of stopping the progression of the disease, after administering the immunomodulating agents, PCL may progress or be induced for reasons currently unknown [183]. Dirk Elston, in his letter discussing the role of dupilumab in CTCL, agrees with our findings. He points out that if a cytokine is upregulated, it does not mean we must down regulate it, contrary to the saying “If it’s wet, dry it. If it’s dry, wet it’’ [184]. Concerning the theory we have previously described, we will now discuss what researchers and medical agencies have found when the aforementioned drugs have been used clinically. We were unable to find any reports on the impact of lebrikizumab, tralokinumab, fezakinumab, and nemolizumab on PCLs in PubMed. Also, we performed the search in the “Drug Safety-related Labeling Changes (SrLC)”—the Federal Drug Agency (FDA) database—and could not find any records on these biologic agents [185]. When searching the European Medicines Agency (EMA) website, found information on tralokinumab (Adtralza), which is authorized for use in the European Union in the treatment of moderate to severe AD [186]. We also found an agreement on the investigation plan for pediatric use of lebrikizumab [187].

Despite a multitude of evidence on potential benefits of using JAKi in the treatment of other lymphoid malignancies, the only JAK inhibitor with reported effects on patients with PCLs is ruxolitinib [188]. Baricitinib, upadacitinib, tofacitinib, and ruxolitinib are authorized for use in the European Union [189,190,191,192]. Abrocitinib was recently positively opinioned for marketing authorization [193]. Prior to 22 October 2021, the only JAK inhibitor registered for AD treatment in the EU is baricitinib (Olumiant) [192]. Interestingly, according to the U.S. Food and Drug Administration Database, baricitinib (Olumiant) and tofacitinib (Xeljanz) may increase risk of developing lymphomas, including those of the skin [194,195]. This warning especially raises the problem of potential increased risk of serious adverse events, including cardiovascular and malignant complication in the treatment of chronic inflammatory conditions [195]. Ruxolitinib and upadacitinib do not have such warnings or precautions in their records because they are not used for treatment of arthritis or other inflammatory conditions [195,196,197]. However, the issue of JAK inhibitor safety in the treatment of AD is raised, since they share the same mechanisms of action [198]. Moreover, currently the only JAK inhibitor that has been studied in a big, four-year surveillance study is tofacitinib [198].

Only dupilumab and ruxolitinib will be discussed in the subsequent paragraph, because to the best of our knowledge, only these drugs are reported to have been administered in the treatment of CTCL misdiagnosed as AD or eczema, CTCL itself, and AD followed by CTCL. These cases are recorded in Table 2.

An expert opinion on the safety of dupilumab shows that it is a safe, well-tolerated drug in AD [214]. Noticeably, CTCLs which occur during treatment with this drug may be unrelated, but a long-term follow-up performed with a large cohort of patients is needed to elucidate this subject [214]. Another opinion describes the cases of MF or SS identified in patients treated with dupilumab and concludes that in a limited subset of patients, this drug might appear to be beneficial [91]. However, generally it should be avoided and in some cases contraindicated for CTCL treatment [91,215]. Our research of the Pubmed database has led us to identify a total of 23 cases in which a PCL and use of dupilumab coexisted [199,200,201,202,203,204,205,206,207,208,209,210,211]. A total of 21 people in this group were above 40 years old [199,200,201,202,203,204,205,206,207,209,210,211]. What may be surprising in the context of our theoretical assumptions is that the most common event in the mentioned group was the progression of the lymphoma, which led to the death of two patients, who progressed to SS [199,200,201,202,203,204,205,206,207,209,210,211]. No clinical improvement of the CTCL was observed four times, whereas the disease course improved in three cases [199,203,206,207,211]. In 16 cases, the original diagnosis was AD or eczema while remaining patients were treated for PCL or mogalizumab-associated rash off-label [199,200,201,202,203,204,205,206,207,209,210,211]. Interestingly, dupilumab appeared to be effective for the treatment of lichenoid reaction associated with mogalizumab in a patient with CD8+ MF [211].

Ruxolitinib, which targets JAK1/JAK2 is used in the treatment of psoriatic arthritis, AD, and several lymphoid malignancies, e.g., myelofibrosis and polycythemia vera [215,216]. Moreover, trials on animal models of hemophagocytic lymphohistiocytosis (HLH) prove this JAK inhibitor to be efficient in the treatment of this condition [217,218]. The genetic abnormalities in the JAK/STAT pathway have been the rationale for therapeutic uses that we discussed earlier. Assuming that JAK inhibitors prove to be effective in the treatment of cutaneous lymphomas, clinicians may feel comfortable administering them if the final diagnosis is difficult to make [215]. These facts led the researchers to administer ruxolitinib to nine patients with PCLs (four MF, three non-specified CTCL, one primary cutaneous anaplastic large cell lymphoma (pcALCL), and one subcutaneous panniculitis-like-T-cell lymphoma (SPTCL)) [212,213]. Some was observed in three cases (one MF, one pcALCL and one STPCL), but the disease course remained stable or worsened in the others [212,213]. Interestingly, despite that five of the seven CTCLs showed the signs of JAK/STAT activation, only one patient whose tumor showed 20% overactivation of pSTAT3 responded to the treatment [213].

The conflicting data revealed in this article seems to be in line with our previous considerations on the safety and danger of the use of biologics in the treatment of psoriasis. Blockage of several mechanisms by which the interleukins act and occur in PCLs should be beneficial in the treatment of the disease. However, dupilumab, in most of patients with lymphoma misdiagnosed as AD or eczema, makes it fully apparent. This drug does not seem to be beneficial for CTCL patients in most cases. Accordingly, despite the JAK/STAT activation, most of the lymphomas did not respond to ruxolitinib. With this paper, we would like to raise awareness to the issue of a development or a misdiagnosis of a cutaneous lymphoma in patients with AD. Especially for patients that are 40 years old or above, the chronic and severe course of AD and the sudden worsening of the symptoms should be considered “red flags” to exclude the potential oncologic risk by taking and carefully verifying the biopsy.

## 3. Materials and Methods

A comprehensive search of the literature using the PubMed (https://pubmed.ncbi.nlm.nih.gov/) electronic database using the search queries “(IL-4 and cutaneous lymphoma) OR (IL-4 and mycosis fungoides)”, “IL-22 and cutaneous lymphoma”, and “IL-31 and cutaneous lymphoma” was performed in the second week of August 2021, from the database inception to the 14th of August 2021. Further research using the queries “(dupilumab and lymphoma)”, “(fezakinumab and cutaneous lymphoma) or (fezakinumab and mycosis fungoides)”, “(lebrikizumab and cutaneous lymphoma) or (lebrikizumab and mycosis fungoides)”, “(tralokinumab and cutaneous lymphoma) or (tralokinumab and mycosis fungoides)”, “(baricitinib and cutaneous lymphoma) or (baricitinib and mycosis fungoides)”, “(ruxolitinib and cutaneous lymphoma) or (ruxolitinib and mycosis fungoides)”, “(upadacitinib and cutaneous lymphoma) or (upadacitinib and mycosis fungoides)”, and “(jak inhibitor and cutaneous lymphoma) or (jak inhibitor and mycosis fungoides)” was performed in the third week of August 2021, from the database inception to the 25th of August 2021 and a “((jak) OR (stat)) AND (cutaneous lymphoma)” search was performed in the second week of September 2021, from the database inception to the 11th of September 2021. After the initial search, titles and abstracts were screened for the inclusion and exclusion criteria. Based on title and abstract analysis, we included articles concerning the role of IL-4, IL-13, IL-22, IL-31, JAK/STAT, and biologic drugs affecting cytokine profiles and JAK inhibitors on PCLs. At this step, we excluded records not related to the topic, non-English manuscripts, personal opinions, and duplicates. The remaining were qualified as eligible for full-text reading. After reading the full manuscripts, some were excluded (not relevant, not original, and not providing information concerning earlier mentioned cytokines, pathways, and new drugs’ impact on PCLs). Finally, additional relevant, eligible records identified through a references search were included, in which information on the effect of PCL microenvironmental influence on the specific lymphoma subtypes were included. Concentration of cytokines in the biopsies and in the blood of the patients, genetic alterations concerning genes linked to the featured subject, the possible effects of interleukins, pathways, and administration of the agents blocking them in the clone cells were analyzed and summarized.

## 4. Conclusions

IL-4, IL-13, IL-22, and IL-31 are detectable in the lesions and sera of patients suffering from PCL. The JAK-STAT pathway has an established role in the oncogenesis of this tumor. We summarized the effects of these cytokines in the course of cutaneous lymphomas. We have shown that IL-4, IL-13, and activation of certain JAKs and STATs to be crucial in the development of the tumoral microenvironment as well as in the progression of the disease. IL-22 and IL-31, however, are not as important in the pathogenesis of PCL as they are in AD. Based on the publications, we have also described the effect of dupilumab and ruxolitinib in the PCL patients misdiagnosed as AD, in PCL itself or AD itself and with PCL in follow up during treatment (coexistence of two diseases). The progression of the lymphoma was observed in most of cases. Thus, we would like to highlight, that in case of severe AD or eczema, especially in a case of rapid evolution of the symptoms in an older individual, it is necessary to perform a biopsy from the skin lesion. Subsequently, a close pathological examination to exclude the possibility of PCL misdiagnosis/evolution should be performed. However, as the authors, we would like to mention that we should not avoid dupilumab in severe AD, especially concerning the portfolio of other systemic drugs used in AD, such as cyclosporine A, methotrexate, and azathioprine, all with known immunosuppressive potential. We realize the necessity of further research concerning the role of IL-22 and IL-31 in PCL. Furthermore, the new biologic and small molecule drugs should be used carefully in the treatment of AD in order not to worsen the course of the cutaneous lymphomas.

## Figures and Tables

**Table 1:** Clinical and immunological similarities between atopic dermatitis (AD) and cutaneous T-cell lymphoma (CTCL).

Similarities	Atopic Dermatitis	Cutaneous T-Cell Lymphoma
Eosinophilia	Often present	May be present in the advanced stage
Immunoglobulin E (IgE)	Often elevated	May be elevated in the advanced stage
Lactate dehydrogenase (LDH)	May be elevated	Severity marker of MF/SS
Soluble interleukin receptor 2 (sIL-2R)	May be elevated	Severity marker of MF/SS
Th-2 microenvironment activation	Always present	Present in the advanced stage
Levels of filaggrin	Significantly lowered	May be significantly lowered
Transepidermal water loss (TEWL)	Significantly lowered	May be significantly lowered
Levels of antimicrobial peptides (AMPs)	Significantly lowered	Significantly lowered
Colonization of *S. aureus*	80% of patients	50–60% of patients

**Table 2:** Cutaneous T-cell lymphoma cases treated with dupilumab or ruxolitinib. We have updated the table continuing the results by doctor Sugaya [91].

Drug	Age (Years)	Sex	Pre-Diagnosis	Final Diagnosis	Response to Treatment	Death	Reference
Dupilumab	58	M	AD	MF	Progression of MF	No	[199]
Dupilumab	64	M	AD	SS	Progression of SS	No	[200]
Dupilumab	51	F	AD	MF	Progression of MF	No	[201]
Dupilumab	64	M	AD	CTCL-NOS	Progression of erythroderma	No	[202]
Dupilumab	72	M	AD	MF	Progression of MF	No	[202]
Dupilumab	59	F	AD	MF and AD	Progression of MF	No	[202]
Dupilumab	40	F	AD	MF	Progression of MF	No	[202]
Dupilumab	67	M	MF	SS	Progression of SS	Yes	[202]
Dupilumab	58	M	MF	SS	Progression of SS	Yes	[202]
Dupilumab	77	F	MF	SS	Progression of SS	No	[202]
Dupilumab	61	M	Eczema	MF	Progression of MF	No	[203]
Dupilumab	52	M	Eczema	MF	No clinical improvement	No	[203]
Dupilumab	60	F	Eczema	MF	No clinical improvement	No	[203]
Dupilumab	68	M	SS and AD	SS and AD	Improvement in SS and AD	No	[204]
Dupilumab	37	F	Eczema	SS	Progression of SS	No	[205]
Dupilumab	55	M	MF and AD	MF and AD	Improvement of MF and AD	No	[205]
Dupilumab	74	F	SS	SS	Improvement of SS	No	[206]
Dupilumab	48	F	AD	SS and AD	No clinical improvement	No	[207]
Dupilumab	40	F	AD	MF	Progression of MF	No	[208]
Dupilumab	43	M	AD	MF and AD	Progression of MF	No	[209]
Dupilumab	48	F	AD	MF	Progression of MF	No	[210]
Dupilumab	55	M	AD	MF	Progression of MF	No	[210]
Dupilumab	26	M	MF	MF	No clinical improvement	No	[211]
Ruxolitinib	13	M	HLH	HLH and SPTCL	Improvement of SPTCL and HLH	No	[212]
Ruxolitinib	NS	NS	MF	MF	Progression of MF	No	[213]
Ruxolitinib	NS	NS	CTCL	CTCL	No clinical improvement/Stable disease	No	[213]
Ruxolitinib	NS	NS	CTCL	CTCL	Progression of CTCL	No	[213]
Ruxolitinib	NS	NS	CTCL	CTCL	Progression of CTCL	No	[213]
Ruxolitinib	NS	NS	MF	MF	Progression of MF	No	[213]
Ruxolitinib	NS	NS	MF	MF	No clinical improvement/Stable disease	No	[213]
Ruxolitinib	NS	NS	MF	MF	Improvement of MF/Partial remission	No	[213]
Ruxolitinib	NS	NS	pcALCL	pcALCL	Improvement of MF/Complete response	No	[213]

Abbreviations: NS: not specified; M: male; F: female; MF: mycosis fungoides; AD: atopic dermatitis; CTCL: cutaneous t-cell lymphoma; pcALCL: primary cutaneous anaplastic large-cell lymphoma; SS: Sézary Syndrome; HLH: hemophagocytic lymphohistiocytosis; SPTCL: subcutaneous panniculitis-like T-cell lymphoma; CTCL-NOS: CTCL-not otherwise specified.

## Data Availability

All the data can be found in the PubMed database—https://pubmed.ncbi.nlm.nih.gov/ or under the links cited of cited websites.
